# Pinyin Is an Effective Proxy for Early Screening for Mandarin-Speaking Children at Risk of Reading Disorders

**DOI:** 10.3389/fpsyg.2020.00327

**Published:** 2020-02-26

**Authors:** Shaowei Ma, Xiumei Zhang, Hunter Hatfield, Wen-Hua Wei

**Affiliations:** ^1^School of Foreign Languages, Langfang Teachers University, Langfang, China; ^2^Department of English and Linguistics, University of Otago, Dunedin, New Zealand; ^3^School of Teacher Education, College of Education, University of Canterbury, Christchurch, New Zealand; ^4^Department of Women’s and Children’s Health, Dunedin School of Medicine, University of Otago, Dunedin, New Zealand

**Keywords:** Chinese reading, dyslexia, early screening, morphological awareness, phonological awareness, Pinyin, reading disorder

## Abstract

Reading disorders (RD) are common and complex neuropsychological conditions associated with decoding printed words and/or reading comprehension. Early identification of children at risk of RD is critical to allow timely interventions before mental suffering and reading impairment take place. Chinese is a unique medium for studying RD because of extra efforts required in reading acquisition of characters based on meaning rather than phonology. Pinyin, an alphabetic coding system mapping Mandarin sounds to characters, is important to develop oral language skills and a promising candidate for early screening for RD. In this pilot study, we used a cohort of 100 students (50 each in Grades 1 and 2) to derive novel profiles of applying Pinyin to identify early schoolers at risk of RD. Each student had comprehensive reading related measures in two consecutive years, including Pinyin reading and reading comprehension tested in the first and second year, respectively. We showed that Pinyin reading was mainly determined by phonological awareness, was well developed in Grade 1 and the top predictor of reading comprehension (explaining ∼30% of variance, *p* < 1.0e-05). Further, students who performed poorly in Pinyin reading [e.g. 1 standard deviation (SD) below the average, counting 14% in Grade 1 and 10% in Grade 2], tended to perform poorly in future reading comprehension tests, including all four individuals in Grade 1 (two out of three in Grade 2) who scored 1.5 SDs below the average. Pinyin is therefore an effective proxy for early screening for Mandarin-speaking children at risk of RD.

## Introduction

Reading disorders (RD) are conditions occurring in learning to decode printed words (i.e. developmental dyslexia) and/or in learning to comprehend text (i.e. reading comprehension impairment) (Cutting et al., 2013; Hulme and Snowling, 2016; Snowling et al., 2019). Children with RD tend to have poor reading comprehension and thus low educational attainment and poor employment prospects (Hulme and Snowling, 2016; Snowling et al., 2019). Deciphering RD has been a long-standing challenge for over a century (Anderson and Meier-Hedde, 2001; Peterson and Pennington, 2015; Stein, 2018). This is largely owing to the high complexities in RD and the lack of coherent definitions across disciplines (Snowling, 2012; Snowling and Hulme, 2012; Stein, 2018). In essence, reading is a cognitive process of mapping letters/words to the sounds they represent in the brain. Hence RD can be considered as symptoms of neuropsychological disorders (Guerrini and Dobyns, 2014). With recent advances in many fields (e.g. neuroimaging), important progress has been made to understand causal mechanisms underlying the highly frequent RD (Pugh and Verhoeven, 2018; Facoetti et al., 2019). While mounting evidence indicate multiple causal links with RD, further work is required to validate these hypothesized links (Gori et al., 2016; Facoetti et al., 2019).

Early identification of children at risk of reading failures is critical to allow timely interventions before the children suffer from potential downward spiral of underachievement, lowered self-esteem and poor motivation (Snowling, 2013). Indeed, during infancy and early childhood, human brains undergo fast development of spatial and temporal architecture and brain functions crucial for future performance (Li et al., 2018). Early practices in the United Kingdom (Fawcett et al., 1998) and United States (Pennington and Lefly, 2001) indicate that pre-school screening tests of RD are feasible, leading to implementation of an “Early Years Foundation Profile Stage” in the United Kingdom (Snowling, 2013) and “Multi-Tier System of Supports” in the United States (Shepley and Grisham-Brown, 2019). These exercises together render a clear view that early oral language difficulties are strong predictors of later RD (Hulme and Snowling, 2016). Early screening for RD appears to be feasible in non-English language systems including Chinese (McBride-Chang et al., 2008, 2011; Pugh and Verhoeven, 2018).

The Chinese language is believed to be a unique medium for studying RD (Tan et al., 2005; McBride et al., 2018). First, Chinese is a morpheme-based logographic system where each character is based on meaning rather than phonology and thus requires not only phonological but also morphological awareness (MA) for reading acquisition (Tan et al., 2005; Pan et al., 2016). Second, differences in the functional neurology (Tan et al., 2001; Zhao et al., 2017) and the genetic associations with reading were observed in Chinese populations (DeMille et al., 2018; Liu et al., 2019). Third, the RD trajectories appear different in Chinese populations (McBride et al., 2018). Nevertheless, cautions are recommended when interpreting results of RD studies from different Chinese communities where teaching systems, social settings for learning to read and RD diagnostic criteria are substantially different (McBride et al., 2018). For example, the estimated prevalence of dyslexia was 9.7% in Hong Kong (Chan et al., 2007) but ∼4% in mainland China (Liu et al., 2016; Zhao et al., 2016).

One typical difference is the introduction of Pinyin (meaning “spell sound”), a phonological coding system using Roman alphabet letters and four lexical tones to indicate the pronunciation of logographic characters, in mainland China but not Hong Kong (Wang et al., 2014; McBride et al., 2018). Pinyin is known to have multifold advantages in promoting Chinese reading: (a) using simple alphabetic transcripts to represent the sounds of Chinese characters; (b) bridging the spoken form with the written forms for each Chinese character and acting as a self-learning tool benefiting both new and experienced readers; (c) facilitating recognition of new characters through sublexical phonology (e.g. tone and syllable awareness); and (d) promoting memorizing and retrieving logographic characters via stable phonological cues (Lin et al., 2010; Wang et al., 2014; Ding et al., 2018). Given these advantages, Pinyin can be taught informally to kindergarteners as young as 3 years old and appears to be a good predictor of future Chinese reading performance (Lin et al., 2010; McBride-Chang et al., 2012; Yin and McBride, 2018). These factors together render Pinyin a potential valuable approach for early screening for children at risk of RD.

However, Pinyin is formally taught only in the first year of primary schools in mainland China (Wang et al., 2014; McBride et al., 2018). Several issues need to be addressed before any Pinyin screening applications become possible in Mandarin-speaking populations. One key issue is the lack of informative profiles illustrating how Pinyin reading proficiency post formal teaching may interplay with Chinese reading skills in the initial school years and particularly predict future reading failures. Previous evidence showed that poor readers in higher grades (e.g. Grade 4) did suffer more from Pinyin reading difficulties than normal readers (Yin and Weekes, 2003; Ding et al., 2015). This pilot study is therefore conducted to fill the information gap by re-analyzing the data generated from a project studying early Chinese reading development (Ma, 2016). We report characteristics of Pinyin reading measured after the formal Pinyin training in a study cohort and derive profiles of using Pinyin reading to identify children at risk of RD in early grades.

## Materials and Methods

### Participants

One hundred children (50 in Grade 1 and 50 in Grade 2) from a state-funded mainstream school in Langfang (near Beijing, China) participated in this study, each with data measured in two consecutive academic years. All participants are native speakers of Mandarin – the official and instruction language in mainland China, where children normally start primary school around 7 years old and receive the full Pinyin training before learning to read Chinese characters. Pinyin teaching normally takes the first 12 weeks to cover onsets, rhymes, and lexical tones and spelling rules. The Pinyin phonetic symbols are continuously presented alongside Chinese characters in textbooks until Grade 3, and are provided only when new characters are introduced. None of the participants had obvious behavioral or emotional problems according to their class teachers. All participants were tested for receptive vocabulary using the Chinese version of Peabody Picture Vocabulary Test-Revised and appeared to have normal verbal intelligence (Lu and Liu, 1998).

### Procedure and Measures

All reading related measures included in the study were administered to the participants individually by a trained examiner in a quiet room during the second semester of each academic year.

We included eight measures for phonological awareness (PA), four for MA, and four for rapid automatized naming (RAN) and Pinyin reading, which were measured for each participant in their first year of the study entry (i.e. school year 1 for Grades 1 and year 2 for Grade 2). We also included reading comprehension as the second outcome measure that was measured for each participant 1 year later after the study entry. We briefly describe these measures below. The full details and the summary statistics of these measures are available in Supplementary Note 1 and Supplementary Table 1, respectively.

#### Reading Outcome Measures

##### Pinyin reading

A novel measure where children were asked to read out 50 one-syllable and 25 two-syllable words all in Pinyin scripts and scored for each syllable pronounced correctly. The maximum score is 75.

##### Reading comprehension

This measure was designed following the model by Elbeheri et al. (2011) where children were asked to answer 36 multiple-choice questions silently in 15 min and scored for each correct answer. The maximum score is 36.

#### Phonological Awareness Measures

Commonly used tests of phonological identification (Bradley and Bryant, 1983), deletion (McBride-Chang and Ho, 2000) and production (Chung et al., 2008) were employed to assess abilities to manipulate sounds at the syllabic, onset-rime and phonemic levels. For each measure, two practice tests were given prior to 15 formal tests to ensure sufficient understanding of how to perform the task properly, and children were scored for each correct answer in the formal test where no feedback was provided. The maximum score is 15.

##### Initial sound identification/deletion

Children were asked to orally identify the odd initial sound from a set of three Chinese syllables provided with a same tone, or to orally delete the initial sound from a syllable provided.

##### Final sound identification/deletion

Children were asked to orally identify the odd final sound from a set of three Chinese syllables provided with a same tone, or to orally delete the final sound from a syllable provided.

##### Rhyme detection/production

Children were asked to orally identify the odd rhyme from a set of three Chinese syllables provided with a same tone, or to orally produce a real syllable with the same rhyme as that shared in the two Chinese syllables provided.

##### Tone detection

Children were asked to orally identify the odd tone from a set of three Chinese syllables (differ in both onsets and rimes) provided.

##### Syllable identification

Children were asked to orally identify the odd syllable from a set of three two-syllable Chinese words provided.

#### Morphological Awareness Measures

Commonly used tests of homograph discrimination (Ku and Anderson, 2003) and production (Shu et al., 2006), homophone discrimination and production (Wenling et al., 2002) were employed to assess understanding of meaning and structure of compound words. For each measure, two practice tests were given prior to 15 formal tests to ensure sufficient understanding of how to perform the task properly, and children were scored for each correct answer in the formal test where no feedback was provided. The maximum score is 15.

##### Homograph discrimination/production

Children were asked to orally identify the odd item with a unique meaning in the common morpheme shared in three two-character Chinese words provided, or to produce a two-character Chinese word with a different meaning from that in a common character shared by a pair of two-character Chinese words provided.

##### Homophone discrimination/production

Children were asked to orally identify the odd item with a unique meaning in the common homophonic morpheme shared by three two-character Chinese words provided, or to produce a two-character Chinese word with a different meaning from that in the homophonic syllable shared by a pair of two-character Chinese words provided.

#### Rapid Automatized Naming Measures

Four existing measures (Elbeheri et al., 2011; Liao et al., 2015) were adopted to assess ability to rapidly name graphological or non-graphological objects including ***digit naming*** testing of five single-digit integer numbers, ***picture naming*** testing of six color pictures of common objects, ***character naming*** testing of five simple Chinese characters, and ***Pinyin letter naming*** testing of five Pinyin letters. Only objects that are familiar to children were chosen in the tests. For each measure, one practice test was given prior to the formal test to ensure sufficient understanding of how to perform the task properly, and then children were instructed to read each object as fast and accurately as possible in the formal test and the time taken in pronouncing the given objects was recorded.

### Statistical Analysis

All statistical analyses were conducted in R (R Core Team, 2018) using packages available from https://cran.r-project.org/. In addition to summary statistics, pairwise Pearson correlations between the reading related measures were calculated using the *cor()* function and visualized using the *corrplot()* function in the ***corrplot*** package. Scatter plots were generated using the *ggscatter()* function in the ***corrplot*** package. Two additional sets of analyses were conducted to quantify impact of the cognitive measures on Pinyin reading and reading comprehension and to generate profiles of mock screening tests based on Pinyin reading.

#### Factor Analysis and Linear Regression

Exploratory factor analyses of measures in each of PA, MA, and RAN categories were performed, respectively, using the ***psych*** package in steps: (1) using *VSS()* to explore how many factors to be reduced from the given pairwise correlations; (2) using the *factanal()* to perform factor analysis by setting the reduced number of factors and the score method as “Bartlett”; and (3) storing the resultant factor scores for further analyses. Linear regressions and subsequent analyses of variance were performed to assess relative impact of PA, MA, and RAN factors on Pinyin reading and reading comprehension using *lm()* and *anova()*, respectively. Variance inflation factor and tolerance of multicollinearity were assessed for each regression model using *ols_vif_tol()* in the ***olsrr*** package. The R scripts used in the analyses and relevant details are available in Supplementary Note 2.

#### Mock Screening Test Analysis

Using a threshold of 1 SD below the average in Pinyin reading, mock screening tests were performed for students in Grades 1 and 2, respectively. Assuming those performed 1.5 SDs below the average in reading comprehension to be the “true” cases with RD, the screening test results were analyzed to derive (1) a screening out rate, calculated as the number of screened out divided by the total number of samples, (2) screen out true positive rate, calculated as the number of cases screened out divided by the total number of cases, and (3) screen out false positive rate, calculated as the number of non-cases divided by the total number of individuals screened out.

## Results

All reading related measures included in the study were informative with clear variations (Supplementary Table 1). These measures were often highly correlated and their correlations varied with grades (Supplementary Figure 1). Pinyin reading was strongly correlated with almost every reading related measure in Grade 1, and the correlations remained strong in Grade 2 except for those with the MA measures, indicating a great potential of Pinyin for early screening for RD in school students. Most PA measures were strongly correlated with other measures relatively consistently across grades, whereas the correlations between the MA and the RAN measures were generally weak/moderate (Supplementary Figure 1).

**FIGURE 1:**
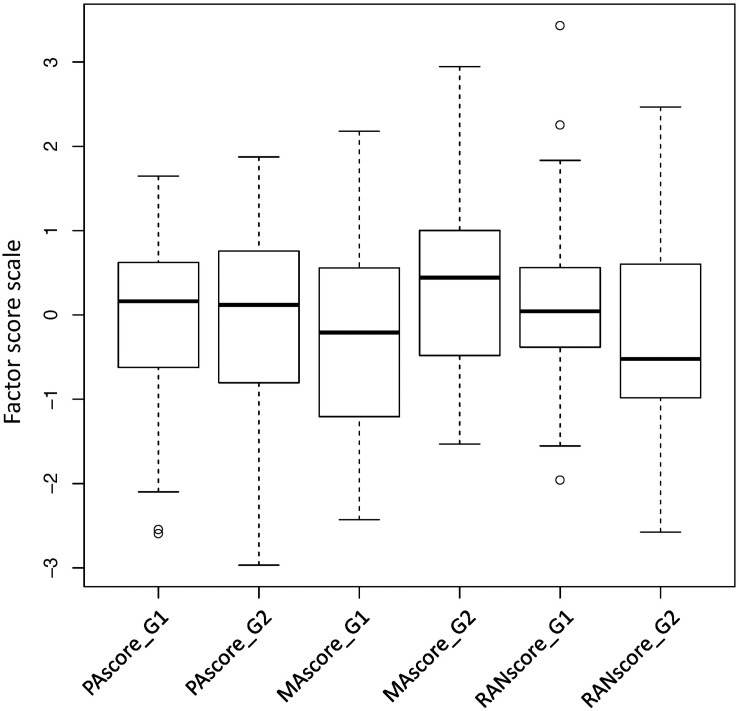
Box plots of factor scores derived from phonological, morphological and rapid naming measures in Grades 1 and 2, respectively. PAscore_G1(2): phonological awareness factor score in Grade 1(2); MAscore: morphological awareness factor score in Grade 1(2); RANscore: rapid naming factor score in Grade 1(2).

Exploratory factor analyses were performed for each category of reading related measures to simplify the complex correlation structures, and resulted in 1-factor solutions for every category and standardized factor scores named as PAscore, MAscore, and RANscore, respectively (Supplementary Note 2). The distributions of the factor scores showed clearly that, compared with the counterparts in Grade 1, MAscore and RANscore were substantially improved in Grade 2 whereas PAscore remained relatively stable (Figure 1), suggesting PA measures were probably well developed in Grade 1.

Variance analyses showed that PAscore was indeed the top predictor of Pinyin reading, contributing 71.8% of the total variance (*p* = 1.4e-15) in Grade 1 and 47% (*p* = 1.9e-09) in Grade 2 (Table 1). RANscore was also a significant predictor of Pinyin reading but only in Grade 2, contributing 15.7% of the total variance (*p* = 7.9e-05). For reading comprehension, when fitting Pinyin reading as the first covariate followed by the factor scores, Pinyin reading was the top predictor explaining 33.9% of the total variance (*p* = 1.2e-06) in Grade 1 and 29.9% (*p* = 7.8e-06) in Grade 2 (Table 1). Despite the collinearity with Pinyin reading, PAscore remained the second predictor of reading comprehension, contributing 10.4% of the total variance (*p* = 0.003) in Grade 1 and 7.5% (*p* = 0.015) in Grade 2, whereas RANscore again contributed significantly only in Grade 2 (9.6%, *p* = 0.006). When fitting Pinyin reading as the last covariate after the factor scores, Pinyin reading became the least predictor explaining little additional variance of reading comprehension in either Grade as expected. These results jointly suggest that Pinyin reading statistically is a good proxy for the PA, MA and RAN measures (Supplementary Figure 2).

**TABLE 1:** Variance explained by each attribute in reading outcome*.

		Pinyin reading				Reading comprehension		
		
	Grade 1		Grade 2		Grade 1		Grade 2	
				
Attribute	Variance %	*p*	Variance %	*p*	Variance %	*p*	Variance %	*p*
Pinyin reading	n/a	n/a	n/a	n/a	33.9%	1.2e–06	29.9%	7.8e–06
PAscore	71.8%	1.4e–15	47.0%	1.9e–09	10.4%	0.003	7.5%	0.015
MAscore	0.0%	0.787	0.8%	0.343	6.8%	0.016	1.8%	0.224
RANscore	1.9%	0.060	15.7%	7.9e–05	0.4%	0.569	9.6%	0.006

* Gender is not important in any models; age was significant (*p* = 0.009) in the Grade 1 model of Pinyin reading only, accounting for 3.7% of total variance.

**FIGURE 2:**
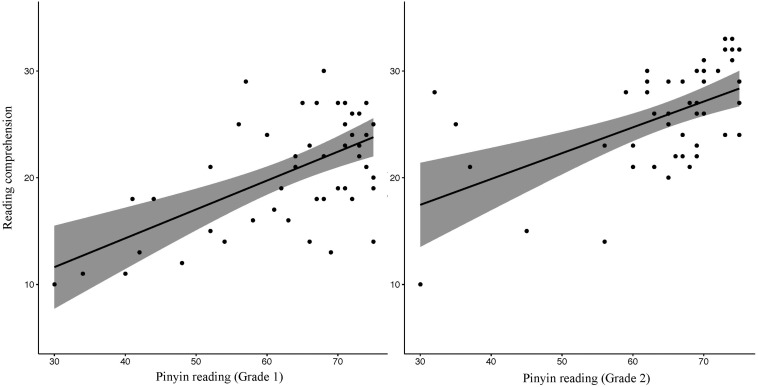
Scatter plots of Pinyin reading against reading comprehension in Grades 1 and 2, respectively. For each plot, data points represented each as a black dot; regression trend represented as a black line and confidence intervals represented in shaded areas.

We then visually examined the distributions of Pinyin reading against reading comprehension (Figure 2). While the performances in the two outcome readings corresponded in general, the data points at the bottom left (poor performers in both) were much sparser than those at the top right (good performers in both) in each plot, indicating that most students underwent healthy development of Chinese reading and Pinyin reading could indeed pick up poor readers, particularly in Grade 1 where students were just in their second year of school when reading comprehension was measured and thus were still within the early stage of reading development as evidenced by wide variation in reading comprehension. In contrast, in Grade 2, data points clearly clustered in two groups by either reading outcome, but Pinyin reading became less indicative with outliers in both directions (Figure 2) possibly because of other reading developments (e.g. RAN skills) in these older students.

Assuming students with 1.5 SDs below the average of reading comprehension as RD cases, we further performed mock screening tests using a threshold of 1 SD below the average of Pinyin reading (Table 2). The mock test in Grade 1 identified 7 (14%) students as being at risk of RD including all four predefined RD cases, and thus had 100% screen out true positive rate in early detection of RD. Of the remaining three classified as false positives, S32 could well be a RD case because the student had a reading comprehension of 13 slightly above the threshold of 12.6 but had the poorest RANscore and the second poorest PAscore (Supplementary Table 2). The mock test in Grade 2 identified 5 (10%) students including only 2 of the 3 predefined RD cases, where the missed RD case S57 was the oldest student who had the second poorest RANscore and all three false positives (i.e. S66, S71, and S81) had a poor RANscore coincidently (Supplementary Table 3).

**TABLE 2:** Pinyin mock screening test results.

Item	Grade 1	Grade 2
Total samples with data	50	49
Threshold of Pinyin reading	52.0	53.3
Threshold of reading comprehension	12.6	18.45
Number of RD cases	4	3
Total screened out	7	5
RD cases screened out	4	2
Screening out rate	14%	10%
Screen out true positive rate	100%	67%
Screen out false positive rate	43%	60%

## Discussion

In this pilot study, we ascertained the feasibility of using Pinyin reading to screen cohorts of early schoolers for individuals at risk of RD. Within the data collected, all predefined poor readers were screened out by simply applying a threshold of 1 SD below the average of Pinyin reading in Grade 1. The screening tests identified less than 15% of students as being at risk for RD, which is a strong first step in identifying those in need for early intervention and full assessment. Furthermore, implementation of the Pinyin screening is convenient and cost effective given Pinyin training is compulsory for every new primary school student in mainland China. Ideally, such implementations could happen immediately after the Pinyin teaching in order to maximize the window of effective interventions under the current teaching system.

Applying Pinyin early screening in Grade 1 is also endorsed by the facts that Pinyin reading was mostly determined by PA and both were well developed in Grade 1 with only small changes of means in Grade 2 (Figure 1 and Supplementary Table 1). In contrast, morphological awareness had a late onset of development to learn Chinese characters and meanings (McBride et al., 2018), and thus had limited values in predicting either Pinyin reading or reading comprehension at this early school stage (Table 1). However, the influence of MAscore might have been offset partially by PAscore in the regression model of reading comprehension because of their correlations (Table 1 and Supplementary Figure 1) and the unique mediation relationship between syllable awareness (phonological) and morphological awareness in Chinese (Pan et al., 2016). Similarly, rapid naming skills had limited values in predicting reading outcome in Grade 1 but became important in Grade 2, as shown in previous studies (Lervag and Hulme, 2009; Liao et al., 2015).

Can Pinyin early screening be implemented at the preschool stage? The answer is probably yes given the reasons above and the successful examples of English early screening in the United Kingdom and United States, each also relying heavily on PA (Snowling, 2013; Shepley and Grisham-Brown, 2019). However, further investigations of Pinyin applications at the kindergarten stage are needed to generate comprehensive and coherent evidence for policy makers in mainland China, as showed in the development of the English examples (Snowling, 2013). One obvious discrepancy is that Pinyin reading of early schoolers (i.e. 7 or 8 years old) explained ∼30% of the reading comprehension variance (Table 1), whereas Pinyin measures (e.g. Pinyin invented spelling) of kindergarteners could explain less than 10% of the variance of future Chinese reading (McBride-Chang et al., 2012; Yin and McBride, 2018). One main reason for the discrepancy could be that only simple Pinyin skills can be taught informally in kindergartens. Therefore, it is essential to develop standardized procedures for Pinyin teaching and assessment for kindergarteners.

Although only a small sample of an ordinary school in China was used in this pilot study, the observed rates of students at risk of RD (i.e. 8 and 6% in Grades 1 and 2, respectively, Table 2) however, are in line with the hypothesis that dyslexia could be less prevalent in Mandarin-speaking communities as reported previously (Dai et al., 2016; Liu et al., 2016; Zhao et al., 2016) than in Hong Kong (9.7%) (Chan et al., 2007) where Pinyin is not used in Chinese teaching. In addition to different diagnostic criteria used, differences in education systems and special social settings for learning in Chinese populations could also explain the discrepancy in the reported prevalence of dyslexia. For example, Confucianism-based motivation leads to preschool education and private trainings (e.g. music, painting) commonly adopted in mainland China, which may virtually act as effective interventions and thus reduce the prevalence of dyslexia often measured at school age (Dai et al., 2016; Pan et al., 2017; McBride et al., 2018). Besides, Pinyin learning could be another hidden intervention since the key cognitive-linguistic skills for learning Chinese (e.g. phonological sensitivity) may be initially integrated in Pinyin training (Wang et al., 2014; McBride et al., 2018). Further investigations of differences in dyslexia prevalence across the Chinese communities and any additional roles of Pinyin (Ding et al., 2018; Chen et al., 2019) are therefore warranted.

Successful early screening for children at risk of RD could boost genetic studies that are limited mainly by small samples available and heterogeneity in phenotyping across ethnic communities (DeMille et al., 2018; Liu et al., 2019). With small at-risk groups, it is economically feasible to apply new but expensive technologies such as functional and structural magnetic resonance imaging (Skeide et al., 2015, 2016; Kraft et al., 2016) to improve diagnoses as well as characterization of intermediate features (e.g. working-memory and hearing) highly associated with RD (Mannel et al., 2015; Neef et al., 2017). These together will greatly increase the number of well-defined RD cases and consequently the power of genetic association studies, which in turn will enable genetic prediction of the RD risk (Muller et al., 2016). Furthermore, these could promote cross-population dissection of the genetic mechanisms underlying RD by meta-analyzing data derived from Chinese and European samples and eventually identify any Chinese-specific genetic variants (Rosenberg et al., 2010; Liu et al., 2019).

Nonetheless, cautions are recommended when interpreting the results of this pilot study that is limited by small samples and hypothesized statistical analyses without actual diagnosis of RD in any samples. Using the threshold of 1 SD (instead of 1.5 SD) below the average of Pinyin reading was to ensure all RD cases were found in early screening for Grade 1. The estimates of screen out true positive rate and percentages of students at risk of RD based on such a small sample size can only be indicative at most. Large and well-designed longitudinal cohort studies are needed to generate accurate profiles for Pinyin screening tests at both the school and pre-school stage. Such studies will simultaneously benefit genetic epidemiology studies of dyslexia in China.

## Conclusion

Pinyin is an effective proxy for early screening for Mandarin-speaking children at risk of RD.

## Data Availability Statement

The datasets generated for this study are available on request to the corresponding author.

## Ethics Statement

Participation in this study was voluntary and with parental/guardian written informed consent. Ethics approval for this study was provided by the Ethics Committee of the University of Canterbury (reference number: 2012/56/ERHEC).

## Author Contributions

SM, HH, and W-HW designed the study. SM and W-HW analyzed the data and prepared the draft and final manuscript. SM collected the data and performed the study. XZ assisted the data collection and contributed to the manuscript writing. HH contributed to the data analysis and manuscript writing.

## Conflict of Interest

The authors declare that the research was conducted in the absence of any commercial or financial relationships that could be construed as a potential conflict of interest.
