# Supplementary material for: Pinyin Is an Effective Proxy for Early Screening for Mandarin-Speaking Children at Risk of Reading Disorders
Source: Front Psychol. 2020 Feb 26;11:327. doi: 10.3389/fpsyg.2020.00327 (PMC7055296; doi:10.3389/fpsyg.2020.00327)
Supplement: Supplementary file 1 [file Table_1.DOCX]

Supplementary Material

# Supplementary Figures and Tables

## Supplementary Figures

**Supplementary Figure 1.** Pairwise correlations between measures taken in Grade 1 (left) and Grade 2 (right). The measures are displayed in the order of categories of phonological awareness (syllable identification, initial sound identification, initial sound deletion, final sound identification, final sound deletion, rhyme identification, rhyme production, tone identification), morphological awareness (homophone discrimination, homograph discrimination, homophone production, homograph production), rapid naming (digit naming, pinyin letter naming, character naming, picture naming) and outcome (Pinyin reading, Reading comprehension); each correlation coefficient represented by a colored pie where the size and color are indicated by the color panel on the right showing positive correlation in blue and negative correlation in red; on the diagonal are the correlation with self for each measure, i.e. correlation coefficient is 1 and thus in the full pie size with dark blue color; only significant correlations (i.e. either less than -0.28 or greater than 0.28 at the level of p < 0.05 given the fixed sample size of 50) are plotted and insignificant correlations are set blank; black connected rectangular aligning with morphological measures added for convenience of viewing.

**Supplementary Figure 2.** Scatter plots of Pinyin reading against factor scores of phonological awareness (top panel), morphological awareness (middle panel) and rapid naming (bottom panel) in Grade 1 (left) and Grade 2 (right). In each plot, data points represented each as a black dot; regression trend represented as a black line and confidence intervals represented in shaded areas.

## Supplementary Tables

**Supplementary Table 1**: Summary information of reading measures used in the study*

|  |  | Grade 1 |  | Grade 2 |  |
| --- | --- | --- | --- | --- | --- |
| measure | brief name | mean | SD | mean | SD |
| syllable identification | sitTOT | 12.7 | 2.2 | 13.1 | 2.0 |
| initial sound identification | isitTOT | 9.9 | 3.2 | 10.3 | 3.4 |
| initial sound deletion | isdtTOT | 12.2 | 2.7 | 12.4 | 3.2 |
| final sound identification | fsitTOT | 7.4 | 2.7 | 7.5 | 2.7 |
| final sound deletion | fsdtTOT | 6.5 | 3.0 | 6.5 | 3.3 |
| rhyme identification | ritTOT | 8.6 | 3.2 | 8.8 | 3.1 |
| rhyme production | rptTOT | 9.9 | 2.4 | 10.3 | 2.7 |
| tone identification | titTOT | 11.9 | 2.8 | 12.1 | 2.7 |
| homophone discrimination | hpdtTOT | 5.1 | 3.2 | 7.7 | 2.8 |
| homograph discrimination | hgdtTOT | 6.3 | 3.6 | 7.3 | 4.3 |
| homophone production | hpptTOT | 6.3 | 3.0 | 7.8 | 3.1 |
| homograph production | hgptTOT | 6.5 | 3.1 | 7.4 | 2.9 |
| digit naming | dnt | 26.3 | 5.3 | 25.9 | 6.8 |
| pinyin letter naming | plnt | 45.5 | 11.5 | 43.3 | 11.2 |
| character naming | cnt | 36.3 | 10.3 | 31.1 | 7.5 |
| picture naming | pnt | 35.1 | 7.1 | 32.1 | 7.8 |
| Pinyin reading | prtc | 63.5 | 11.5 | 64.3 | 11.0 |
| Reading comprehension | rcompT2 | 20.7 | 5.4 | 25.8 | 4.9 |

*: 50 participants in each of Grade1 and Grade2, which were further measured for rcompT2 one year later where one participant dropped off

**Supplementary Table 2**: Students identified as at risk of reading disabilities and/or true cases in the Pinyin mock screening test in Grade 1*

| sample | age | Pinyin reading | Reading comprehension | PAscore | MAscore | RANscore | at_risk | RD_case |
| --- | --- | --- | --- | --- | --- | --- | --- | --- |
| S4 | 84 | 48 | 12 | -1.81 | -1.92 | 0.95 | yes | yes |
| S15 | 96 | 30 | 10 | -2.59 | -2.43 | 1.74 | yes | yes |
| S20 | 88 | 41 | 18 | -1.36 | 0.41 | 0.91 | yes | no |
| S21 | 98 | 40 | 11 | -1.71 | -1.79 | 1.38 | yes | yes |
| S24 | 88 | 34 | 11 | -2.10 | -2.14 | 2.25 | yes | yes |
| S32 | 92 | 42 | 13 | -2.55 | 0.24 | 3.43 | yes | no |
| S47 | 82 | 44 | 18 | -0.90 | -0.68 | 1.34 | yes | no |

*: at_risk: Pinyin reading is less than 52.0; RD_case: Reading comprehension is less than 12.6

**Supplementary Table 3**: Students identified as at risk of reading disabilities and/or true cases in the Pinyin mock screening test in Grade 2*

| sample | age | Pinyin reading | Reading comprehension | PAscore | MAscore | RANscore | at_risk | RD_case |
| --- | --- | --- | --- | --- | --- | --- | --- | --- |
| S4 | 84 | 48 | 12 | -1.81 | -1.92 | 0.95 | yes | yes |
| S15 | 96 | 30 | 10 | -2.59 | -2.43 | 1.74 | yes | yes |
| S21 | 98 | 40 | 11 | -1.71 | -1.79 | 1.38 | yes | yes |
| S24 | 88 | 34 | 11 | -2.10 | -2.14 | 2.25 | yes | yes |
| S32 | 92 | 42 | 13 | -2.55 | 0.24 | 3.43 | yes | no |
| S47 | 82 | 44 | 18 | -0.90 | -0.68 | 1.34 | yes | no |

*: at_risk: Pinyin reading is less than 53.3; RD_case: Reading comprehension is less than 18.45

# Supplementary Note 1: Full description of the measures included

***Phonological awareness (PA) measures***

Eight measures were developed to assess abilities to manipulate sounds at the syllabic, onset-rime and phonemic levels, including tests of identification (Bradley and Bryant, 1983), deletion (McBride-Chang and Ho, 2000) and production (Chung et al., 2008) commonly used in both English and Chinese literature. For each measure, two practice tests were given prior to 15 formal tests to ensure sufficient understanding of how to perform the task properly; no feedback was provided during the formal tests; children scored for each correct answer with a maximum of 15.

*Initial sound identification/deletion*: to assess ability to discriminate the initial sounds of Chinese syllables or to segment the initial onset of a Chinese syllable. In the identification test, children were orally presented with a set of three syllables with the same tone (labelled as either 1, or 2 or 3 or 4, and highlighted in red in the following examples marked by square brackets) and asked to identify the odd one with a different initial sound. For example, after listening to [ma3], [mai3], [da3], children were asked to choose the syllable that had the different initial sound from the others. The correct answer, in this case, is [da3].

In the deletion test, Children were asked to delete orally the initial sound from a syllable presented by the assessor in each test. For example, after listening to a syllable [zhang1], children were asked to say [zhang1] and say it again without saying the initial sound [zh]. The right answer, in this case, is [ang]. The Cronbach's alpha coefficient for the current sample was 0.713.

*Final sound identification/deletion*: novel measures developed to assess awareness of single phonemes at the end of syllables in Mandarin Chinese. In the identification test, children were orally presented with a set of three syllables with the same tone and were asked to identify the odd one with a different final (single) sound. For example, after listening to three syllables [san3], [ben3], [mang3], children were asked to identify the syllable that ends with a different (single) sound. The right answer, in this case, is [mang3].

In the deletion test, Children were asked to delete orally the final (single) sound from a heard syllable presented aloud by the assessor. For example, after listening to a syllable [huan1], children were asked to say [huan1] and say it again without the final /n/ sound. The right answer, in this case, is [hua1]. The Cronbach's alpha coefficient for the current sample was 0.686.

*Rhyme detection/production*: to assess phonological awareness at the rhyme level. In the detection test, children were orally presented with three syllables with the same tone and asked to identify the odd one that did not rhyme with the other two. For example, after listening to three syllables [tan1], [ban1], [dun1], children were asked to find the syllable that did not rhyme. The right answer is [dun1]. The odd one out could be in any order.

In the production test, children were provided two Chinese syllables that rhymed (e.g. /fei1/ and /bei1/) and asked to produce another real syllable that rhymed the same as the two provided in 10 seconds (e.g. /lei1/, /fei1/, or /hei1/). The maximum score was 15. The Cronbach's alpha coefficient for the current sample was 0.670.

*Tone detection*: to assess tone detection ability. Children were orally presented with three syllables that differ in both onsets and rimes and asked to identify the odd one with a tone different from that in the other two. For instance, after listening to three syllables [lao2], [tan2], [hai1], children were asked to choose the odd one that had a different tone from the others. The right answer in this case, is [hai1].

*Syllable identification*: to assess ability to identify syllable. Children were orally presented with three two-syllable words and asked to identify the odd one without none syllable shared with the others. For example, after listening to a set of three two-syllable words [dong1tian1], [dong1gua1], [tan2hua4], children were asked to find the word that did not share any syllable with the others. In this case, the right answer is [tan2hua4].

## *Morphological awareness (MA) measures*

Four measures were used to assess understanding of meaning and structure of compound words, including homograph discrimination (Ku and Anderson, 2003) and production (Shu et al., 2006), homophone discrimination (Wenling et al., 2002) and production tests commonly used in Chinese literature. For each measure, two practice tests were given prior to 15 formal tests to ensure sufficient understanding of how to perform the task properly; no feedback was provided during the formal tests; children scored for each correct answer with a maximum score of 15.

*Homograph discrimination/production*: to assess awareness of polysemantic features of Chinese in which one morpheme or character might convey different meanings in different word contexts. Words used in the test were from common oral vocabulary determined by three primary Chinese language teachers. In the discrimination test, children were orally presented with three two-character words sharing a common morpheme and asked to identify the odd one with a different meaning in the common morpheme. For example, after listening to the words 月光 [yue-guang] (moonlight), 月色 [yue-se] (moonbeam), and 年月 [nian-yue] (days), the children were asked to choose the word in which the common part 月 [yue] represented a different meaning from the others. The right answer in this case, is 年月[nian-yue] (days).

In the production test, children were orally presented with a pair of two-character words containing one common character with the same meaning and asked to produce another two-character word with the common character conveying a different meaning. For example, after listening to a pair of two-syllable words 朝阳 [zhao-yang] (the rising sun) and 夕阳 [xi-yang] (the setting sun), children were asked to produce another word with the common character 阳 [yang] conveying different meaning. One correct answer in this case, can be 阳刚 [yang-gang] (masculine). The Cronbach's alpha coefficient for the current sample was 0.803.

*Homophone discrimination/production*: to assess awareness of polyphonic feature of Chinese where characters with the same pronunciations may have different meanings (morphemes) and/or written forms. Words used in the test were from common oral vocabulary determined by three primary Chinese language teachers. In the discrimination test, children were orally presented with three two-character words sharing a homophonic morpheme and asked to identify the odd with a different meaning in the homophonic morpheme. For example, after listening to the words画家 [hua-jia] (painter), 图画 [tu-hua] (picture) and说话 [shuo-hua] (speak), the children were asked to choose the word in which the homophonic morpheme [hua] had a different meaning from the others. The right answer in this case, is说话 [shuo-hua] (speak).

In the production test, children were orally presented with a pair of two-character words sharing one homophonic syllable (sound) and asked to produce another two-character word with the homophonic syllable conveying different meaning. For example, after listening to a pair of two-syllable words树木 [shu-mu] (tree) and 目光 [mu-guang] (eyesight), children were asked to produce another word with the common syllable [mu] conveying a different meaning. One correct answer in this case, can be 沐浴 [mu-yu] (bath).

***Rapid automatic naming (RAN) measures***

Four existing measures (Elbeheri et al., 2011; Liao et al., 2015) were adopted to assess ability to name graphological or nongraphological objects. Only objects that are familiar to children were chosen in the tests. For each measure, one practice test was given prior to the formal test to ensure sufficient understanding of how to perform the task properly; during the formal test children were instructed read each object in a fixed order from beginning to end as fast and accurately as possible and the time used from pronunciation of the first object to pronunciation of the last item was recorded as the result in seconds.

*Digit naming*: test ability of naming 5 single-digit integer numbers. Children were asked to name digits of **2, 4, 5, 7, 9** that were equally distributed randomly by row in a 10 × 5 matrix on a single sheet of paper, with 10 iterations.

*Picture naming*: test ability of naming 6 color pictures of common objects. Children were asked to name color pictures of pen, door, key, rabbit, fish, and house that were equally distributed randomly by row on a single sheet of paper, with 6 iterations.

*Character naming*: test ability of naming 5 simple Chinese characters. Children were asked to name simple and familiar Chinese characters **大, 天, 少, 不, 小** that were distributed randomly by row in a 10 × 5 matrix on a single sheet of paper, with 10 iterations.

*Pinyin letter naming*: test ability of naming 5 Pinyin letters used in the Mandarin phonetic system. Children were asked to name Pinyin letters of **b, p, f, n, l** in Pinyin pronunciation that were distributed randomly by row in a 10 × 5 matrix on a single sheet of paper, with 10 iterations.

***Reading outcome measures***

*Pinyin reading*: a novel measure to assess impact of Pinyin on reading development. Children were asked to read out 50 single syllables and 25 two-syllable words all in Pinyin scripts and scored for each syllable pronounced correctly with a maximum of 75.

*Speed reading comprehension*: to assess reading comprehension fluency using the model by Elbeheri *et al.* (2011). Children were asked to answer 36 multiple-choice questions silently in 15 minutes and scored for each correct answer, with a maximum of 36. The questions were Grade-appropriate, in the format of incomplete sentences or short passages with one missing character or word to be selected from three choices provided. The questions were given with increasing difficulty. The Cronbach's alpha coefficient for the current sample was 0.86. An example is as follows:

在音乐课上，武老师教我们中国的传统乐器，我们都____她讲课，在她的课上，我们不仅获取了更多的中国传统音乐的知识，而且我们在学习过程中得到很多的乐趣。

A． 喜欢 B. 讨厌 C. 害怕 (答案A)

*English translation:*

In music class, Teacher Wu taught us how to play Chinese traditional musical instruments, and we all ___ her teaching. In her class, not only we acquired more knowledge of Chinese classic music, but also we got a lot of fun in learning process.

A. enjoyed B. hated. C. feared (the answer is A)

***Reference***

Bradley, L., and Bryant, P.E. (1983). Categorizing sounds and learning to read—a causal connection. *Nature* 301(5899)**,** 419-421. doi: 10.1038/301419a0.

Chung, K.K.H., McBride-Chang, C., Wong, S.L., Cheung, H., Penney, T.B., and Ho, C.S.H. (2008). The role of visual and auditory temporal processing for Chinese children with developmental dyslexia. *Annals of Dyslexia* 58(1)**,** 15-35. doi: 10.1007/s11881-008-0015-4.

Elbeheri, G., Everatt, J., Mahfoudhi, A., Abu Al-Diyar, M., and Taibah, N. (2011). Orthographic Processing and Reading Comprehension Among Arabic Speaking Mainstream and LD Children. *Dyslexia* 17(2)**,** 123-142. doi: 10.1002/dys.430.

Ku, Y.-M., and Anderson, R.C. (2003). Development of morphological awareness in Chinese and English. *Reading and Writing* 16(5)**,** 399-422. doi: 10.1023/a:1024227231216.

Liao, C.H., Deng, C., Hamilton, J., Lee, C.S., Wei, W., and Georgiou, G.K. (2015). The role of rapid naming in reading development and dyslexia in Chinese. *J Exp Child Psychol* 130**,** 106-122. doi: 10.1016/j.jecp.2014.10.002.

McBride-Chang, C., and Ho, C.S.H. (2000). Developmental issues in Chinese children's character acquisition. *Journal of Educational Psychology* 92(1)**,** 50-55. doi: Doi 10.1037//0022-0663.92.1.50.

Shu, H., McBride-Chang, C., Wu, S., and Liu, H.Y. (2006). Understanding Chinese developmental dyslexia: Morphological awareness as a core cognitive construct. *Journal of Educational Psychology* 98(1)**,** 122-133. doi: 10.1037/0022-0663.98.1.122.

Wenling, L., Anderson, R.C., Nagy, W., and Houcan, Z. (2002). "Facets of Metalinguistic Awareness that Contribute to Chinese Literacy," in *Chinese Children’s Reading Acquisition: Theoretical and Pedagogical Issues,* eds. L. Wenling, J.S. Gaffney & J.L. Packard. (Boston, MA: Springer US), 87-106.

# Supplementary Note 2: Exploratory factor analysis and results

***R scripts used for exploratory factor analyses***

# load packages

require(psych)

library(GPArotation)

require(foreign)

# loading research data object named as paper1short

load("MyWorkData.RData")

# PA section

ic<-c(6:13) # phonological awareness measures

myset<-paper1short[,ic]

items <- names(myset)

# explore indicators of how many factors

nf=5 # assuming 5 to start

psych::VSS(cor(myset[items]), nf, n.obs=nrow(myset), rotate="promax")

# Loadings after rotation.

fitAfterRotation <- factanal(myset[items],

factors = 1, rotation = "promax", scores = "Bartlett")

print(fitAfterRotation$loadings, cutoff = .10, sort = TRUE)

# store resultant standardized score (mean: 0, standard deviation: 1)

paper1short$PAscore <-fitAfterRotation$scores

# MA section

ic<-c(14:17) # morphological awareness measures

myset<-paper1short[,ic]

items <- names(myset)

# explore indicators of how many factors

nf=3 # assuming 5 to start

psych::VSS(cor(myset[items]), nf, n.obs=nrow(myset), rotate="promax")

# Loadings after rotation.

fitAfterRotation <- factanal(myset[items],

factors = 1, rotation = "promax", scores = "Bartlett")

print(fitAfterRotation$loadings, cutoff = .10, sort = TRUE)

# store resultant standardized score (mean: 0, standard deviation: 1)

paper1short$MAscore <-fitAfterRotation$scores

# RAN section

ic<-c(18:21) # rapid naming measures

myset<-paper1short[,ic]

items <- names(myset)

# explore indicators of how many factors

nf=3 # assuming 5 to start

psych::VSS(cor(myset[items]), nf, n.obs=nrow(myset), rotate="promax")

# Loadings after rotation.

fitAfterRotation <- factanal(myset[items],

factors = 1, rotation = "promax", scores = "Bartlett")

print(fitAfterRotation$loadings, cutoff = .10, sort = TRUE)

# store resultant standardized score (mean: 0, standard deviation: 1)

paper1short$RANscore <-fitAfterRotation$scores

save.image("MyWorkData.RData")

***Results of exploratory factor analyses***

# PA section results

Very Simple Structure

Call: vss(x = x, n = n, rotate = rotate, diagonal = diagonal,fm = fm, n.obs = n.obs, plot = plot, title = title, use = use, cor = cor)

VSS complexity 1 achieves a maximimum of 0.89 with 1 factors

VSS complexity 2 achieves a maximimum of 0.75 with 2 factors

The Velicer MAP achieves a minimum of 0.06 with 1 factors

BIC achieves a minimum of NA with 2 factors

Sample Size adjusted BIC achieves a minimum of NA with 4 factors

> fitAfterRotation

Call:

factanal(x = myset[items], factors = 1, scores = "Bartlett", rotation = "promax")

Uniquenesses:

sitTOT isitTOT isdtTOT fsitTOT fsdtTOT ritTOT rptTOT titTOT

0.345 0.217 0.526 0.671 0.680 0.401 0.583 0.498

Loadings:

Factor1

sitTOT 0.809

isitTOT 0.885

isdtTOT 0.688

fsitTOT 0.573

fsdtTOT 0.566

ritTOT 0.774

rptTOT 0.646

titTOT 0.709

Factor1

SS loadings 4.078

Proportion Var 0.510

Test of the hypothesis that 1 factor is sufficient.

The chi square statistic is 71.26 on 20 degrees of freedom.

The p-value is 1.13e-07

# MA section results

Very Simple Structure

Call: vss(x = x, n = n, rotate = rotate, diagonal = diagonal,fm = fm, n.obs = n.obs, plot = plot, title = title, use = use, cor = cor)

VSS complexity 1 achieves a maximimum of 0.85 with 1 factors

VSS complexity 2 achieves a maximimum of 0.71 with 2 factors

The Velicer MAP achieves a minimum of 0.13 with 1 factors

BIC achieves a minimum of NA with 1 factors

Sample Size adjusted BIC achieves a minimum of NA with 1 factors

> fitAfterRotation

Call:

factanal(x = myset[items], factors = 1, scores = "Bartlett", rotation = "promax")

Uniquenesses:

hpdtTOT hgdtTOT hpptTOT hgptTOT

0.710 0.380 0.431 0.431

Loadings:

Factor1

hpdtTOT 0.539

hgdtTOT 0.788

hpptTOT 0.754

hgptTOT 0.754

Factor1

SS loadings 2.049

Proportion Var 0.512

Test of the hypothesis that 1 factor is sufficient.

The chi square statistic is 8.47 on 2 degrees of freedom.

The p-value is 0.0144

# RAN section results

Very Simple Structure

Call: vss(x = x, n = n, rotate = rotate, diagonal = diagonal,fm = fm, n.obs = n.obs, plot = plot, title = title, use = use, cor = cor)

VSS complexity 1 achieves a maximimum of 0.82 with 1 factors

VSS complexity 2 achieves a maximimum of 0.65 with 2 factors

The Velicer MAP achieves a minimum of 0.11 with 1 factors

BIC achieves a minimum of NA with 1 factors

Sample Size adjusted BIC achieves a minimum of NA with 1 factors

> fitAfterRotation

Call:

factanal(x = myset[items], factors = 1, scores = "Bartlett", rotation = "promax")

Uniquenesses:

dnt plnt cnt pnt

0.327 0.490 0.624 0.677

Loadings:

Factor1

dnt 0.821

plnt 0.714

cnt 0.614

pnt 0.568

Factor1

SS loadings 1.882

Proportion Var 0.471

Test of the hypothesis that 1 factor is sufficient.

The chi square statistic is 0.62 on 2 degrees of freedom.

The p-value is 0.732
